# The Cognitive Neurology of Bilingualism in the Age of Globalization

**DOI:** 10.1155/2014/536727

**Published:** 2014-05-08

**Authors:** Jubin Abutalebi, Brendan S. Weekes

**Affiliations:** ^1^Department of Speech and Hearing Sciences, The University of Hong Kong, Hong Kong; ^2^Faculty of Psychology, University San Raffaele, Via Olgettina 58, 20132 Milan, Italy

As globalization advances, more people become bilingual or multilingual. Indeed, in the age of globalization, where people can connect through Internet to someone else across the globe, where the mass media provides information from around the globe, and where migration means multilingual and multicultural societies, the necessity of speaking more than a single language becomes more evident as never before. Some languages like English, French, and Spanish for cultural and political reasons are widespread because of their international use for communication, tourism, and trade. Some languages like Mandarin are emergent and are most likely about to substitute one or two of the aforementioned world languages in the future. As a matter of fact, modern world is becoming more bilingual and modern behavioural neurology and neuroscience must be prepared to deal with challenging research questions originating from the study of the bilingual brain.

In general, a bilingual speaker may be someone with different levels of proficiency in the two languages, using the two languages in different contexts or learning a new language due to educational requirements, immigration, or other business and life demands. By this definition, a bilingual individual is not only necessarily someone who has acquired both languages from birth, or early in life, but also that one who learns a second language (L2) later in life. The different contexts and circumstances of L2 acquisition have important effects upon the cerebral organization of multiple languages. Moreover, having acquired more than one language, the bilingual or multilingual speaker may eventually encounter potential conflicts between languages, such as how to speak in one language while avoiding potential intrusions from the other. Problems like these are resolved by the intervention of a neurocognitive mechanism unique to bilinguals, that is, language control [1].

In the past, a lot of misleading information has circulated about the eventual cognitive effects of bilingualism such as bilingualism as being detrimental to the development of cognitive skills in children and that infants exposed simultaneously to two languages would suffer from an incomplete language acquisition. Nowadays, scientists have proven the contrary to be true. Researchers have clearly demonstrated that early bilingualism (i.e., if both languages are learned from early on in life) leads to cognitive advantages over lifespan. Bilinguals when compared to monolinguals are faster in information processing and conflict resolution in nonverbal tasks [2, 3]. Strikingly, these effects are already present in bilingual infants. Seven-month-old bilingual infants are able to more efficiently switch their attention in a nonlinguistic task than monolinguals [4], and, at 18 months old, they appear to have a more developed memory generalization processes [5].

Related to these behavioural advantages is also the recent discovery that bilingualism induces neuroplastic changes in the brain. Bilingualism as compared to monolingualism induces experience-related structural changes (i.e., in terms of increased grey or white matter density) in several brain areas such as the frontal lobes [6], left inferior parietal lobule [7, 8], the anterior cingulate cortex (ACC) [9], and the subcortical structures such as the left caudate [10] and putamen [11]. These areas are part of the executive control network and this may explain why bilinguals usually have a cognitive advantage in executive control tasks over monolinguals [2].

As to the reasons behind these neurocognitive advantages, we should consider the following two: First, bilinguals usually get less exposure and use of each of their languages when compared to the single language of monolinguals. Second, bilinguals need to monitor their language production system in order to avoid unwanted intrusion from the language not in use. The consequence of these two factors is a more demanding language processing situation that relies heavily upon the intervention of cognitive control mechanism when speaking. Because of the continuous use of these cognitive control mechanisms, the neural networks subserving cognitive control in bilinguals become more tuned and, hence, may be utilized more efficiently also for nonverbal cognitive conflict such as nonlinguistic information processing and conflict resolution. In other words, the bilingual experience strengthens the executive control network and it is reasonable to believe that bilingual individuals create more connections between the single areas of this network following the rules of Hebbian dynamics. Having more connections, in turn, is the most plausible interpretation for explaining recent and compelling findings that the bilingual brain is more resistant against cognitive decline [12, 13]. Bilingualism as such would accordingly act as a cognitive reserve and as a neuroprotective factor against aging. Indeed, recent studies have shown that elderly bilinguals outperform monolinguals in executive control tasks and appear to have a 4-5-year onset delay of behavioral symptoms associated with neurodegenerative diseases such dementia as compared to monolinguals [13, 14].

As exciting as all these recent discoveries on bilingualism seem, modern globalized societies are also confronted with the management of some pathologies that are unique to bilingual and multilingual speakers such as bilingual aphasia which is the main focus of the present special issue. The reader should keep in mind that the incidence of bilingual aphasia is likely to increase and become a clinical issue of primary importance in the field of cognitive neurology because, as stated above, modern society is becoming more and more bilingual and multilingual and because the average age of the aging population is expected to increase in the future. With this inevitable increase in our bilingual caseloads comes the need to determine effective and efficient assessment and treatment strategies that take into consideration the unique needs and skills of bilingual versus monolingual patients. However, at present, we still lack causal explanations of the many features of recovery patterns and there actually is no consensus about the language in which the patient should receive speech therapy. Further advance requires an understanding of the dynamics of recovery [15].

The first contribution to this special issue aims at providing an explanation for the dynamics of recovery. The contribution is authored by Kong, Abutalebi, Lam, and Weekes, and the authors show that impairments in language control are a key feature in bilingual aphasia. As postulated by Paradis [16], problems in language control may explain the various recovery features of bilingual aphasia. However, in their contribution, Kong et al. underline that impairment of language control (i.e., resulting in pathological switching of languages) is paralleled by impairment of domain-general cognitive control. Among similar lines, also the second contribution to this special issue, authored by Dash and Kar, addressed this interesting research question in individuals with bilingual aphasia. However, contrary to Kong et al., the authors suggest the existence of independent but interactive systems for bilingual language control and domain-general cognitive control.

The third contribution is authored by Kiran, Balachandran, and Lucas and has as its main focus the assessment of lexical access deficits in individuals with bilingual aphasia. As the authors rightly claim up to date, there are no clear guidelines on assessment of lexical access in the two languages in individuals with bilingual aphasia. Fundamental lexical retrieval deficits are reported in bilingual individuals with aphasia as compared to healthy bilingual controls, but, most importantly, lexical access deficits are clearly influenced by the degree of language proficiency. This contribution clearly highlights that, although difficult to assess in brain-damaged individuals, the role of language proficiency assessment is central to explain linguistic deficits in individuals with bilingual aphasia.

Finally, we would like to underline that the clinical management of bilingual patients with aphasia raises still several unanswered questions. For instance, we still do not know whether a bilingual individual with aphasia should be rehabilitated only in one language or in both languages. Moreover, it is also not known whether rehabilitation should take place in L1 or rather in L2. These questions may have an immense practical impact and we should be able to provide the solutions to future generations in the globalized world. The fourth contribution of this special issue attempts to provide some solutions. Indeed, Ansaldo and Ghazi Saidi discuss the literature on bilingual aphasia therapy, with a focus on cross-linguistic therapy effects from the treated language to the untreated language. The authors suggest that degree of structural overlap between languages, type of therapy approach, pre- and postmorbid language proficiency profiles, and the status of the cognitive control circuit play a crucial role in the potential for therapy effects from the treated to the untreated language.

The final contribution to this special issue is by S. Ashaie and L. Obler and the authors investigated the effects of variables such as age, education, and bilingualism on confrontation naming in older illiterate and low-educated populations. Interestingly, this study was carried out in the Kashmir highlands and it is all the more impressive that their results revealed that age-related naming declines in a similar fashion to those reported among higher-educated Western populations.

This final contribution is in line with our suggestion for future directions. Indeed, we would like to invite researchers to perform more cross-linguistic studies such as comparing linguistically distant languages such as Indo-European languages versus Ural-Altaic languages, African languages, and even indigenous and isolated languages spoken in more remote areas of the world (Papua Guinea, Amazonia), and so forth. Such studies, apart from providing us with eventual general rules for the organization of the bilingual brain and the eventual management of bilingual aphasia, may provide also a glimpse on the evolution of the human brain and language interface.

We very much hope that the reader will enjoy the comprehensive coverage of the present special issue treating issues inherent to the field of bilingualism from the perspective of cognitive neurology.

*Jubin Abutalebi* *Brendan S. Weekes*
